# Clinical features of autoimmune cerebellar ataxia related to neuronal antibodies

**DOI:** 10.3389/fimmu.2025.1497695

**Published:** 2025-02-13

**Authors:** Yun Cai, Zhijuan Hua, Yanan Chen, Xue Chen, Na Liu, Ting Liu, Qianwen Zhou, Jinghua Li, Weiying Di

**Affiliations:** ^1^ Department of Neurology, Affiliated Hospital of Hebei University, Baoding, China; ^2^ Department of Hepatobiliary Surgery, Affiliated Hospital of Hebei University, Baoding, China

**Keywords:** autoimmune cerebellar ataxia, neuronal antibody, immunotherapy, cerebellar atrophy, prognosis

## Abstract

**Objective:**

This study aimed to investigate the clinical features of neuronal antibodies related to autoimmune cerebellar ataxia (ACA) and to provide guidance for the diagnosis and treatment of this disease.

**Methods:**

Demographic and clinical data were collected from antibody-positive patients with ACA who were admitted to the Department of Neurology, Affiliated Hospital of Hebei University, from January 2018 to February 2023. A retrospective analysis on the clinical manifestations, laboratory examinations, imaging data, treatment, and prognosis was performed.

**Results:**

A total of six patients, including one man and five women, with a median age of 52.5 years, were enrolled in this study. All patients presented with dizziness and gait abnormalities with or without dysarthria. No tumor was found in these patients. Three patients were at the prodromal stage of infection, while one patient exhibited post-ACA fever symptoms and aggravated disease phenotypes. Three patients were positive for anti-glutamate decarboxylase (GAD), while one patient was positive for each of the anti-Tr, anti-mGluR1, and anti-Homer-3 antibodies. The white blood cell (WBC) count and the protein levels of the cerebrospinal fluid (CSF) were increased in four patients, which was in agreement with predominant lymphocytic inflammation. One patient displayed positive signals for CSF-specific oligoclonal proteins. Of the six patients, two were diagnosed with bilateral cerebellar atrophy, and two patients had nonspecific white matter changes. All of the patients received immunotherapy and rehabilitation treatment. Except for the Homer-3-positive patient, the remaining patients showed good prognosis. One patient relapsed.

**Conclusion:**

ACA can be induced or aggravated by infection. The detection of neuronal antibodies is crucial for the precise diagnosis of ACA. Cerebellar system symptoms, such as dizziness, unsteady walking, nystagmus, and dysarthria, are the first and main manifestations of ACA. The head magnetic resonance imaging (MRI) in patients with ACA may be normal or may exhibit abnormalities including cerebellar atrophy and nonspecific white matter changes. Immunotherapy could be effective in most patients with ACA.

## Introduction

Autoimmune cerebellar ataxia (ACA), also known as autoimmune cerebellitis, is a cerebellar disorder mediated by abnormal autoimmune responses. As diagnostic markers of ACA, neuronal antibodies play an important role in its pathogenesis (1). Neuronal antibodies include both neuronal surface antibodies and intracellular antibodies. Neuronal surface antibodies act on specific antigens on synapses or on cell surfaces directly, resulting in nerve conduction dysfunction. When the target antigen is inside the cell, antigen-specific cytotoxic CD8 T cells recognize epitopes from intracellular proteins in the presence of major histocompatibility complex (MHC) class I antigen-presenting molecules, which is considered to be the main factor that leads to neuronal damage (2). There are several etiopathological factors of ACA, including infectious diseases and tumors, which can coexist with autoimmune diseases such as Hashimoto’s thyroiditis. Depending on the diagnosis of malignant tumors, ACA can be categorized into paraneoplastic and non-paraneoplastic types. Patients with certain types of ACA, such as non-paraneoplastic ACA, exhibit better prognosis and treatment outcomes compared with their paraneoplastic counterparts. Therefore, early diagnosis and treatment of patients with ACA are extremely important. ACA is rare and is sometimes difficult to differentiate from neurodegenerative cerebellar ataxia. At the same time, the positive rate of antibody detection in clinical practice is not high. Therefore, at present, ACA is not well understood. In this study, six cases of ACA with positive neuronal antibodies were investigated through analysis of the patients’ demographic and clinical data in order to improve our understanding of this disease.

## Materials and methods

### Patient selection

Six patients with ACA who visited the Department of Neurology of the Affiliated Hospital of Hebei University from January 2018 to February 2023 were enrolled in this study. These patients tested positive for neuronal antibodies and were diagnosed with cerebellar ataxia.

The inclusion criteria were as follows: 1) cerebellar ataxia as the primary symptom; 2) neuronal antibody positivity either in the cerebrospinal fluid (CSF) or in serum samples; 3) patients with complete clinical data in accordance with treatment courses and routine follow-ups.

The exclusion criteria were: 1) absence of cerebellar ataxia as the primary symptom, despite neuronal antibody positivity in the CSF and/or serum; 2) cerebellar ataxia as the main manifestation, but negative for neuronal antibodies in the CSF and serum; and 3) other causes of cerebellar ataxia, such as genetic, metabolic, toxic, and drug-related etiology, were ruled out.

### Neuronal antibody screening

Serum and CSF antibody panels, including ACA [Tr(DNER)/Homer-3/mGluR1/Zic4/ITPR1/ATP1A3/PCA-2/CARPVIII], autoimmune encephalitis (GAD65/NMDAR/AMPAR1/AMPAR2/GABAR/CASPR2/LGI1/IgLON5/DPPX), and aquaporin protein-4 (AQP-4), were examined using both cell- and tissue-based assays (3, 4). Simultaneously, the paraneoplastic antibody profile (Hu/Yo/Ri/PNMA2/CV2/SOX1/amphiphysin) was also examined.

### Clinical data collection

The following data were collected, analyzed, and summarized: sex, age at disease onset, medical history, neurological symptoms, serum autoantibodies and other immune indicators, routine CSF assays, cytology of the CSF, oligoclonal bands, magnetic resonance imaging (MRI) results, immunotherapy, and prognosis. The modified Rankin scale (mRS) and the Scale for the Assessment and Rating of Ataxia (SARA) were used to evaluate the neurological disabilities of patients at admission, at discharge, and at the last follow-up. An mRS ≤2 at the last follow-up was regarded as a good prognosis (5).

### Statistical analysis

The clinical data of six patients were collected, organized using Excel software, and analyzed with SPSS 22.0 statistical software.

## Results

### Demographic and clinical characteristics

A total of six patients, including one man and five women, were analyzed in this study. The age range was 17–67 years, with a median age of 52.5 years. The disease duration (i.e., the interval between cerebellar symptom onset and diagnosis) ranged from 10 days to 10 months (median, 25 days). In this cohort, one patient was complicated with nephrotic syndrome, and three patients were comorbid with diabetes. None of the patients were accompanied with any systemic autoimmune diseases such as Hashimoto’s thyroiditis and Sjogren’s syndrome. Three patients (case nos. 4, 5, and 6) had definite prodromal symptoms, including fever and cough. One patient (case no. 1) developed post-onset fever with aggravation of cerebellar ataxia. There were no similar cases in the family history of these six patients (**Table 1**).

**Table 1:** Clinical data of six patients with autoimmune cerebellar ataxia at first visit.

No.	G/A^a^	Disease duration	Medical history	Prodromal infection symptoms	Tumor	Dizziness	Nystagmus	Limb ataxia	Trunk ataxia	Dysarthria
1	F/46	1 month	N	Fever, cough^b^	N	+	Horizontal^c^	+^c^	+^c^	+^c^
2	F/56	10 months	Diabetes	N	N	+	Vertical	+	+	−
3	F/67	15 days	Diabetes	N	N	+	Horizontal, vertical	+	+	+
4	F/64	1 month	Diabetes	Fever, cough	N	+	Horizontal	+	+	−
5	M/50	20 days	N	Fever, sore throat	N	+	Horizontal	+	+	+
6	F/17	10 days	Nephrotic syndrome	Fever	N	+	Horizontal, vertical	+	−	−

+, positive; −, negative.

A, age; G, gender; M, male; F, female; N, no.

a Age at visit.

b The patient developed these symptoms on the third day of admission.

c Case no. 1 had no such symptoms at the time of admission, but developed these clinical symptoms after fever on the third day of admission.

### Neurological symptoms

Persistent dizziness, limb ataxia, and gait abnormalities with or without dysarthria were the primary neurological symptoms in all the patients. Patient no. 2 had left limb ataxia on admission, who relapsed with bilateral limb ataxia 2 years later. The other five patients had bilateral limb ataxia on admission. Five patients had trunk ataxia (83.33%) and three patients had dysarthria (50%), and all of the patients had central nystagmus (100%). To date, no tumor has been detected in any of these six patients, as described in **Table 1**. On admission, the mRS scores of these patients ranged from 3 to 4 points, while the SARA scores ranged from 16 to 26 points (**Table 2**).

**Table 2:** Treatment and follow-up of six patients with autoimmune cerebellar ataxia.

No.	First-line immunotherapy	mRS/SARA on admission	mRS/SARA at discharge	Long-term Immunotherapy and oral duration^a^	Relapse	Follow-up time (years)	mRS/SARA at last follow-up
1	IVMP, IVIg	4/26	2/14	MMF: 0.5 g bid, 1.5 years	N	6	0/4
2	IVMP, IVIg	3/24	2/15	N	Y^b^	5	1/8
3	IVMP, IVIg	4/26	3/18	MMF: 0.5 g bid, 1 year	N	2.5	1/9
4	IVMP, IVIg	4/20	2/13	MMF: 0.5 g bid, 1.2 year	N	1.25	1/7
5	IVMP, IVIg	4/22	3/16	MMF: 0.5 g bid, 1.5 years	N	4	2/12
6	IVMP, IVIg	3/16	3/15	MMF: 0.5 g bid, 1 year	N	1	3/14

IVMP, intravenous methylprednisolone; IVIg, intravenous immunoglobulin; MMF, mycophenolate mofetil; Y, yes; N, no; mRS, modified Rankin scale; SARA, Scale for the Assessment and Rating of Ataxia.

a Except for case no. 6, the other five patients stopped long-term immunotherapy with MMF.

b Due to relapse after 2 years, case no. 2 was administered immunotherapy with IVIg and IVMP plus MMF orally for 1.5 years.

### Laboratory and imaging characteristics

#### Neuronal antibody screening

Neuronal antibodies were detected in six patients: five patients were positive for both serum and CSF samples, while one patient was positive for the serum sample only due to the absence of a CSF sample. There were one anti-Tr, three anti-GAD65, one anti-mGluR1, and one anti-Homer-3-positive cases in this cohort. However, all of the patients were negative for both AQP-4 and paraneoplastic antibodies.

#### Serum immune indicators

Examination of the serum immune indicators revealed that all patients were negative for anti-thyroglobulin (TG) antibodies, anti-thyroid peroxidase (TPO) antibodies, anti-neutrophil cytoplasmic antibodies (ANCA), antinuclear antibody (ANA), anti-cardiolipin antibodies (ACA), autoantibodies, and immunoglobulin (Ig) antibodies.

#### CSF analysis

The white blood cell (WBC) count and the CSF protein level were increased in four patients (case nos. 1, 4, 5, and 6), in accordance with predominant lymphocytic inflammation. Only one patient (case no. 4) was positive for CSF-specific oligoclonal bands.

#### MRI characteristics

**Figure 1:**
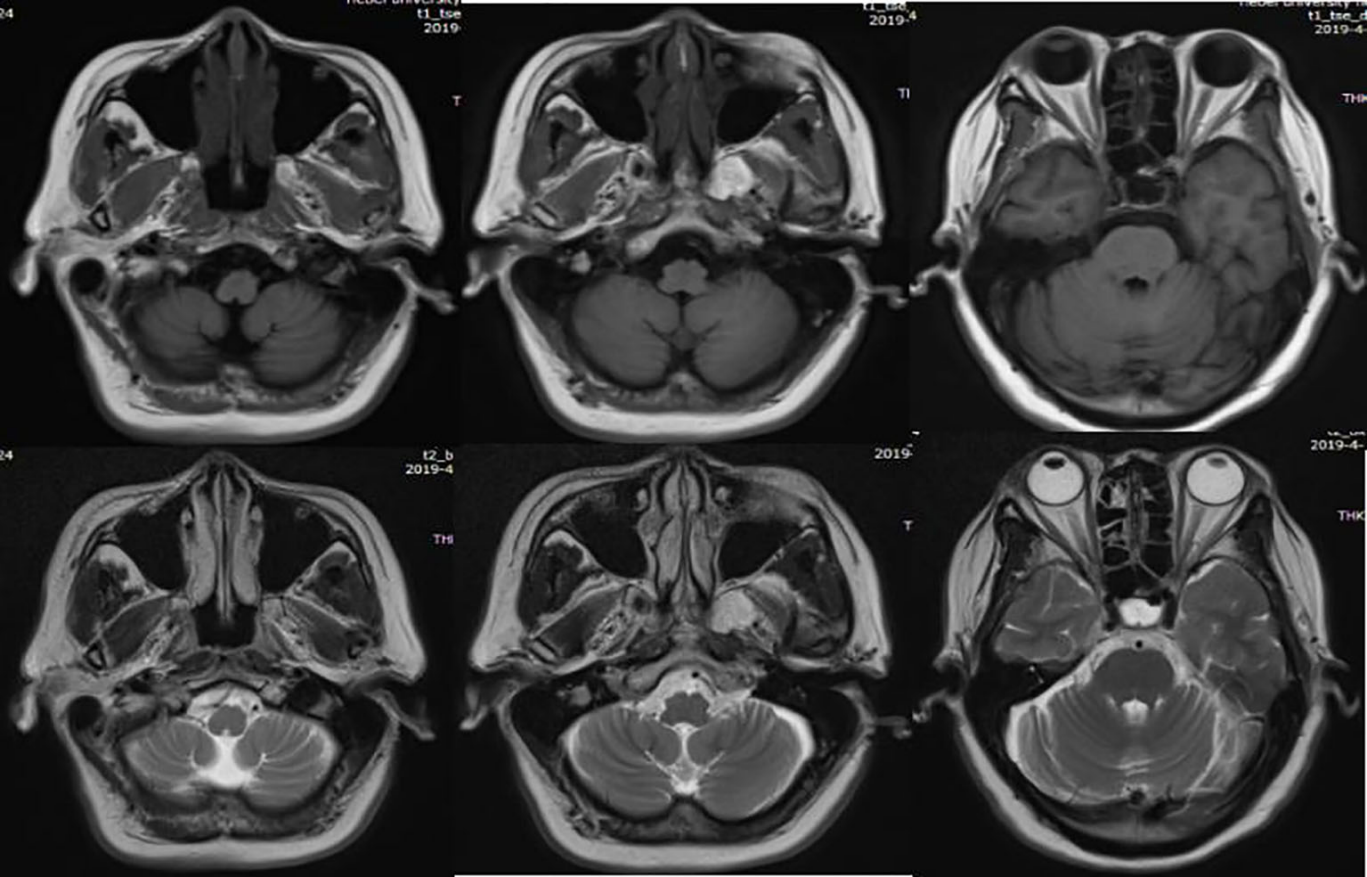
Patient no. 2 showing bilateral cerebellar atrophy in MR.

**Figure 2:**
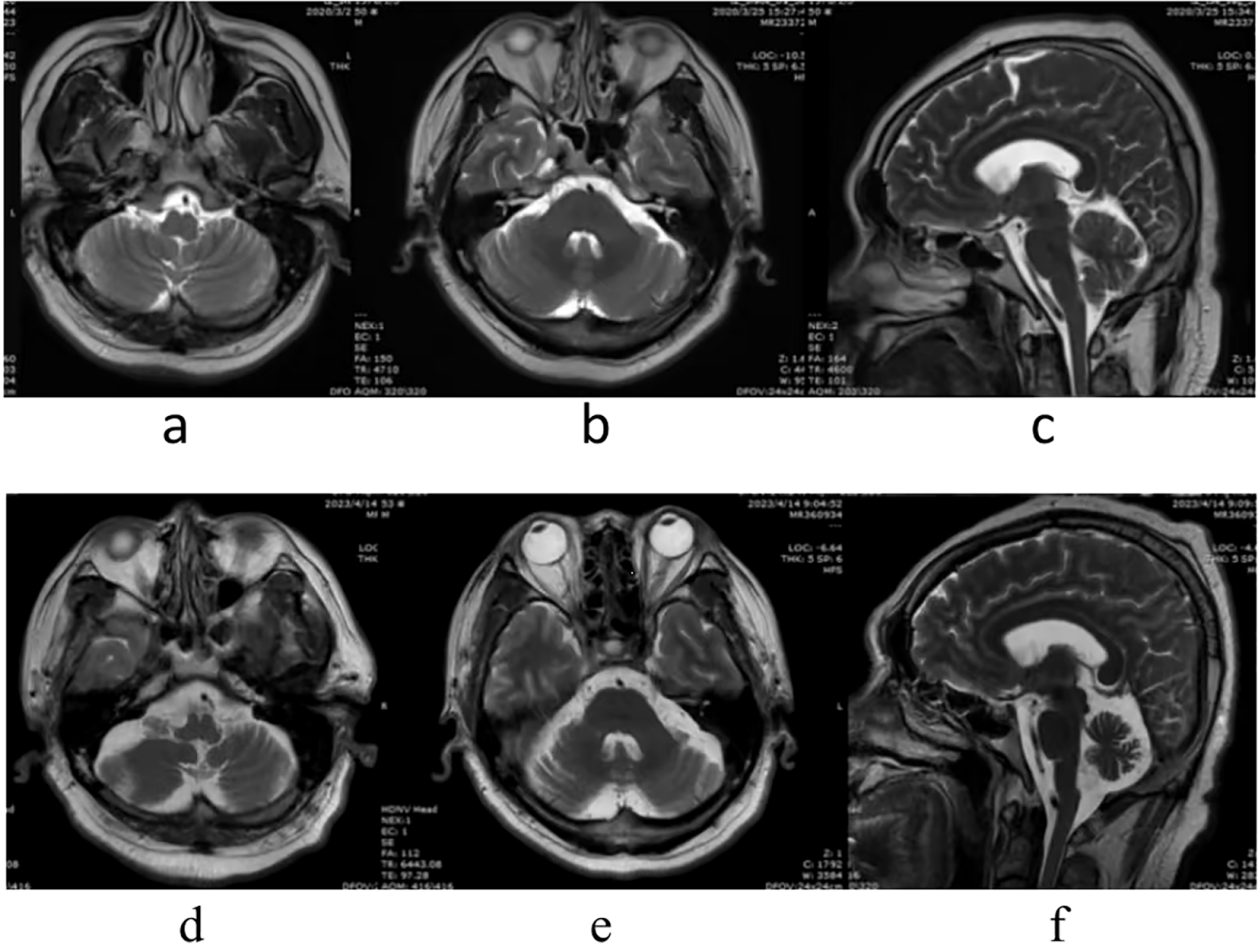
Patient no. 5 had no abnormality on admission, in March 25, 2020 **(A–C)**. However, bilateral cerebellar atrophy was found during follow-up, in April 20, 2023 **(D–F)**.

Bilateral cerebellar atrophy was found in two patients: case no. 2 had bilateral cerebellar atrophy symptoms on admission (**Figure 1**), while case no. 5 was diagnosed with bilateral cerebellar atrophy during follow-up examinations in 2023 (**Figure 2**). Two patients (case nos. 3 and 4) presented with nonspecific white matter changes. The remaining patients had no obvious abnormalities.

#### Tumor screening

Computed tomography (CT) examinations of the chest, abdomen, and pelvic regions and screening of tumor markers were completed during the hospital stay of all patients. At the follow-up, tumor screening, including chest, abdominal, and pelvic CT, tumor marker screening, thyroid and lymph node ultrasonography, breast ultrasonography or mammography for the female patients, and urogenital ultrasonography for the male patient, was performed once every 6 months, with gastrointestinal endoscopy once a year. No tumor has been detected in the patients to date (**Table 3**).

**Table 3:** Examination results of six patients with autoimmune cerebellar ataxia.

No.	CSF pressure (mmH_2_O)	CSF-OB	CSF-Pr (g/L)	CSF-WBC (10^6^/L)	CSF-glucose (mmol/L)	CSF-chloride (mmol/L)	CSF cytology	S- Neuronal antibodies	CSF- Neuronal antibodies	Head MRI
1	205	−	0.6	170	3.4	127	Predominant lymphocytic inflammation	Tr 1:32	Tr 1:32	Normal
2	150	−	0.32	0	4.6	123	Normal	GAD65 1:320	GAD65 1:320	Bilateral cerebellar atrophy
3	60	−	0.43	2	4.1	120	Normal	GAD65 1:100	GAD65 1:320	Nonspecific white matter changes
4	80	+	1.83	110	4.3	116	Predominant lymphocytic inflammation	GAD65 1:20	GAD65 1:10	Nonspecific white matter changes
5	230	−	0.54	190	3.6	124	Predominant lymphocytic inflammation	mGluR1 1:100	NA	Normal
6	170	−	0.49	45	3.3	125	Predominant lymphocytic inflammation	Homer3 1:32	Homer3 1:32	Normal

+, positive; −, negative.

CSF, cerebrospinal fluid; OB, oligoclonal bands; Pr, protein; WBC, white blood cell; S, serum; NA, not available.

#### Treatment and prognosis

All patients were treated with intravenous immunoglobulin (IVIg) doses at 0.4 g kg^−1^ day^−1^ for five consecutive days, combined with pulse corticosteroid therapy (methylprednisolone 1,000 mg/day for 3 days, followed by 500 mg/day for 3 days, 250 mg/day for 3 days, and 125 mg/day for 3 days). After completion of the treatment, the symptoms of cerebellar ataxia were improved in five patients, except for case no. 6. After pulse corticosteroid therapy, all of the six patients were tapered to oral prednisone (1 mg kg^−1^ day^−1^), the dose of which was reduced by 5 mg every 2 weeks (the total course of treatment was approximately 6 months). Mycophenolate mofetil (MMF) was prescribed to five patients as long-term immunotherapy. Case no. 2 was not treated with long-term immunotherapy drugs; instead, this patient was administered a repeated course of a combined regimen of IVIg and methylprednisolone plus MMF orally due to a relapse after 2 years of the first course. The condition of the patient improved after the second course. Except for case no. 6, all of the other patients achieved good prognosis (mRS ≤2) within 6 months (**Table 2**).

## Discussion

ACA is a common cause of sporadic cerebellar ataxia, which can occur in both children and adults (6). Compared with hereditary ataxia, ACA is more treatable with proper and timely diagnosis and treatments. Depending on the etiopathology, the clinical presentation, and the expression of autoantibodies, ACA can be divided into the following subtypes: primary ACA (PACA), paraneoplastic cerebellar degeneration (PCD), autoimmune encephalitis-associated ACA, anti-glutamate decarboxylase (GAD)-associated cerebellar ataxia, opsoclonus–myoclonus syndrome (OMS), and Miller Fisher syndrome (MFS). Among these, PACA and PCD are the most common pathologies (5, 7). Hongzhi Guan’s team found that, with the development of novel anti-cerebellar Purkinje cell antibodies and the application of tissue-based immune assays in major neural immunology study centers, the technology of neuronal antibody detection has been dramatically improved. In the new version of the diagnostic criteria for PACA in 2022, the importance of neuronal antibody detection was emphasized, and the simultaneous applications of cell- and tissue-based assays were recommended in order to compare and validate the results, which can further improve the accuracy and sensitivity of the tests (5).

The neuronal antibody panel includes both neuronal surface antibodies and intracellular antibodies, which have different pathomechanistic implications (2, 8). In this study, the anti-Tr, anti-GAD65, and anti-Homer-3 antibodies were the intracellular antibodies, while the anti-mGluR1 antibody served as a neuronal surface marker antibody. In general, different neuronal antibodies correspond to specific neurological syndromes. For example, in PCD samples, the anti-Tr antibody mainly binds to the extracellular segment of the transmembrane protein DNER in the cytoplasmic and dendritic regions of Purkinje cells. Hence, patients positive for anti-Tr antibodies often present with simple cerebellar ataxia, although some patients exhibit sensory peripheral neuropathies or limbic encephalitis (9). The anti-GAD65 antibody has a direct pathogenic effect on the onset of ataxia by interfering with the release of GABA and by influencing the regulation of excitability of Purkinje cells. The pathogenicity of anti-GAD65 antibodies is related to the recognized antigenic sites, as different epitopes are associated with different diseases, such as stiff person syndrome, cerebellar ataxia, type I diabetes, and thyroiditis (10). However, the PACA, anti-Homer-3, and anti-mGluR1 antibodies are relatively rare, and their direct roles in the pathogenicity of ACA remain to be investigated further (1). Homer-3, encoded by the *HOMER3* gene, is mainly expressed on the axons of Purkinje cells and is involved in the regulation of Ca^2+^ metabolism through interaction with mGluR1 and IP3R. The anti-Homer-3 antibody can act like an anti-Purkinje cell antibody; therefore, the main disease manifestation of related cases positive for this antibody is cerebellar ataxia (11). As a metabolic glutamate receptor, mGluR1 is highly expressed in the dendrites of cerebrocortical Purkinje cells. mGluR1 plays an important role in the rapid signaling of Purkinje cell dendrites. Anti-mGluR1 antibodies can affect cerebellar function by preventing Purkinje cells from inducing synaptic actions. Recent studies have shown that anti-mGluR1 antibodies can specifically lead to a significant reduction in the total and synaptic mGluR1 levels in cultured neurons without affecting the density of other synaptic proteins, such as postsynaptic dense protein 95 (12). ACA could also have overlapping antibodies. The cohort study by Hongzhi Guan’s team revealed that 12.5% of patients with ACA present autoimmune encephalitis mediated by neuronal surface antibodies, such as anti-NMDAR, anti-CasPR2, anti-DPPX, and anti-GABAR (5). Notably, no such overlapping antibodies were found in any of the patients in this cohort, which might be related to the small number of enrolled cases. Highly specific neuronal antibodies, such as anti-Tr, anti-Yo, anti-Hu, anti-Ri, and anti-MA2 antibodies, in the serum and CSF are the key diagnostic biomarkers of PCD (13). In this study, case no. 1, who was positive for the anti-Tr antibody, was not diagnosed with malignant tumors such as lymphoma even after 6 years of follow-up. In the other five patients, the tumor screening also resulted negative. As PCD might appear earlier than the tumor for months or even years, it is necessary to continuously monitor the occurrence of a tumor in patients with positive PCD antibody (14).

In this study, the initial symptom in all patients was cerebellar ataxia, which presented as persistent dizziness (100%), nystagmus (100%), and limb ataxia (100%). The vestibulocerebellar system is an important part of the balanced triad (vision, proprioception, and the vestibulocerebellar system). The vestibulocerebellum receives nerve impulses from the vestibular organ with regard to the spatial position of the head and head movement, and damage to this structure can lead to balance disorders such as dizziness and gait instability, as well as eye movement disorders and central nystagmus. It has been reported that spontaneous downbeat nystagmus (sDBN) can be caused by a variety of lesions involving the cerebellar flocculus or para-flocculus, including paraneoplastic syndrome and encephalitis, among others. In the early stage of these diseases, sDBN and oscillopsia may be the main symptoms and signs of some patients even in a longer period of time. Electronystagmus can be used to assess vestibulocerebellar system function in patients with inconspicuous nystagmus or those with no obvious lesions on imaging. In this cohort, the limb ataxia was bilateral in most cases, except for case no. 2, who had left limb ataxia on admission and who relapsed with bilateral limb ataxia 2 years later. According to the literature review, ACA cases manifested by unilateral ataxia are rare, and the underlying mechanism is unknown. The clinical characteristics of the cases in this study were related to the inclusion criteria, and it should be noted that cerebellar ataxia is not the only phenotype of ACA. The study by Hongzhi Guan’s team was based on 127 patients with ACA and found that many PACA patients had diverse neurological phenotypes, including cerebellar ataxia as the core symptom and other manifestations such as pyramidal tract sign and peripheral nerve radiculopathy as secondary complications (5).

In this study, all patients were positive for serum neuronal antibodies. Except for case no. 5, whose CSF sample was absent, the other five patients were all positive for corresponding antibodies in their CSF samples. The CSF-specific oligoclonal bands indicate the synthesis of intrathecal Ig, which was positive (16.67%) only in case no. 4. The relationship between the oligoclonal bands and neuronal antibodies in the CSF remains unclear. Liu et al. observed that the positive rate of neuronal antibodies in the CSF was higher in ACA patients with oligoclonal bands, but suggested that the study sample size needs to be expanded for further confirmation (15).

Three patients (case nos. 4, 5, and 6) had prodromal infection symptoms, while case no. 1 developed a fever on day 3 of admission and later showed aggravated cerebellar ataxia symptoms. The WBC count and the CSF protein level were increased in four patients (case nos. 1, 4, 5, and 6). These results suggest that immune-associated cerebellitis may be secondary to infection, but the specific mechanism remains unknown. It has been shown that post-infectious cerebellitis (PIC) is mediated by an abnormal autoimmune process triggered by infection (most commonly chicken pox), which is more common in young children (7). Patients with ACA without definite infections occasionally develop fever and other infection-like prodromal symptoms, similar to patients with anti-mGluR2 and anti-Tr (16, 17). However, it remains unclear how the prodromal symptoms indicate infection and immune activation in patients with ACA (18).

It has been noted that cerebellar atrophy can be observed on the brain MRI in patients with cerebellar ataxia, and the severity of atrophy depends on the duration of the disease (19). Of the six cases, two patients (case nos. 1 and 6) displayed no significant abnormalities in MRI examinations. Case no. 2 had bilateral cerebellar atrophy on admission, and the course of the disease had been 10 months at that time. Case no. 5 developed cerebellar atrophy in the third year of the course of disease. However, the symptoms of both patients (case nos. 2 and 5) were significantly improved after immunotherapy and rehabilitation treatments. Patient no. 6 had normal MRI, but poor treatment outcomes, suggesting that the imaging findings were not parallel to the severity of the clinical symptoms and that dynamic observations are required. The other two patients (case nos. 3 and 4) had nonspecific white matter changes, suggesting that MRI in patients with ACA could show abnormalities other than cerebellar atrophy (20).

Similar to other cerebellar ataxia, the common pathological outcome of ACA is irreversible loss of Purkinje cells. However, Mitoma et al. (19) used physiological methods to monitor cerebellar functional survival indicators, and the results showed that ACA is reversible and treatable in the early stage, suggesting the importance of early diagnosis and treatment. There is no widely accepted definition of relapse in ACA currently; therefore, we referred to that in autoimmune encephalitis as exacerbation of cerebellar ataxia after clinical improvement or stabilization for at least 2 months (21). Relapse is not uncommon in ACA, which can present with an initial relapsing course, often occurring after corticosteroid discontinuation or IVIg treatment, especially in patients with PCD (16, 22). Of the six patients, only patient no. 2 relapsed after the first admission, but improved after treatment with the second course of first-line immunotherapy plus MMF oral doses. It was speculated that the relapse might have been caused by the failure of the patient to tolerate long-term immunotherapy at the first visit. Therefore, based on first-line immunotherapy, a timely and effective long-term immunotherapy is extremely important. Although patient no. 6, who was positive for the anti-Homer3 antibody, had timely treatment and routinely received medications, the outcome of immunotherapy was poor, which might be related to the type of antibody involved. Thus far, only more than 10 cases of ACA with the anti-Homer-3 antibody have been reported worldwide. A number of researchers have summarized these reports and found that, after immunotherapy, only some of the patients’ conditions improved and that even some of the conditions worsened, for which the specific mechanism is still unclear (23).

This study has certain limitations. Firstly, this is a single-center study with a small number of patients aged over 14 years, which could have led to bias in the results. In the future, we will increase the number of study cases and conduct multicenter prospective cohort studies to better describe the clinical features of ACA-associated neuronal antibodies.

## Conclusion

In summary, ACA is a common cause of sporadic cerebellar ataxia and has a certain degree of curability. ACA can be induced or aggravated by infectious diseases. Neuronal antibodies play an important role in the pathogenesis of ACA, and the detection of cerebellar autoantibodies can help in the diagnosis and in guiding the screening of malignant tumors in these patients. Cerebellar symptoms such as persistent dizziness, unsteady walking, central nystagmus, and dysarthria are the initial and major manifestations of ACA. The relationship between CSF-specific oligoclonal bands and the expression of neuronal antibodies in the CSF needs to be further studied with larger samples. In patients with ACA, MRI may be normal or may show certain abnormalities such as cerebellar atrophy and nonspecific white matter changes. Dynamic observations of the imaging findings are required due to these not being parallel to the severity of the clinical symptoms. Early diagnosis and implementation of first-line immunotherapy (often with glucocorticoids and/or IVIg) can improve the neurological symptoms in patients with ACA. Standardized long-term immunotherapy can stabilize the disease and reduce its recurrence. At the same time, the type of antibody may affect the prognosis of patients.

## Data Availability

The original contributions presented in the study are included in the article/supplementary material. Further inquiries can be directed to the corresponding authors.
